# Comparison of visual outcomes after femtosecond laser-assisted LASIK versus flap-off epipolis LASIK for myopia

**DOI:** 10.1186/s12886-020-01579-7

**Published:** 2020-07-29

**Authors:** Junjie Piao, Woong-Joo Whang, Choun-Ki Joo

**Affiliations:** 1 Department of Ophthalmology, Peking Union Medical College Hospital, Chinese Academy of Medical Sciences & Peking Union Medical College, No.1 Shuai fu yuan, Dong cheng District, Beijing, 100730 China; 2 Department of Ophthalmology and Visual Science, College of Medicine, Yeouido St. Mary’s Hospital, The Catholic University of Korea, Seoul, Republic of Korea; 3 Department of Ophthalmology, Catholic Institute for Visual Science, Seoul St. Mary’s Hospital, College of Medicine, The Catholic University of Korea, Seoul, Republic of Korea

**Keywords:** Myopia, Femto-LASIK, Flap-off epi-LASIK, Scheimpflug

## Abstract

**Background:**

This study clinically evaluated the visual outcomes after refractive surgery for myopia using femtosecond laser-assisted in situ keratomileusis (femto-LASIK) and flap-off epipolis LASIK (epi-LASIK).

**Methods:**

In this retrospective case series study, 40 eyes of 27 patients were divided into two groups depending on the technique used for refractive surgery. Femto-LASIK and flap-off epi-LASIK flaps were created using femtosecond laser and Epi-K™ epikeratome, respectively. Uncorrected distance visual acuity (UDVA), corrected distance visual acuity, manifest refraction, corneal asphericity, and corneal higher-order aberrations (HOAs) were assessed pre- and postoperatively.

**Results:**

The improvement in logarithm of the minimum angle of resolution (logMAR) UDVA after refractive surgery was statistically significant for both groups (*P* < 0.001 for all groups); it was significant better in UDVA in femto-LASIK than flap-off epi-LASIK, 0.03 ± 0.06 logMAR (femto-LASIK) and 0.54 ± 0.31 logMAR (flap-off epi-LASIK), at 1 day postoperatively; 0.02 ± 0.05 logMAR (femto-LASIK) and 0.14 ± 0.13 logMAR (flap-off epi-LASIK), at 1 week postoperatively (*P* < 0.001 and *P* = 0.019). With regard to the corneal HOAs, the increment in spherical aberration (Z_4,0_) was greater in flap-off epi-LASIK than femto-LASIK: 0.626 ± 0.232 μm and 0.479 ± 0.139 μm in the front cornea; 0.556 ± 0.227 μm and 0.430 ± 0.137 μm in the total cornea (*P* = 0.016 and *P* = 0.017). However, the back corneal HOA changes did not have a significant effect on the total corneal HOA changes.

**Conclusion:**

Femto-LASIK yielded better early visual outcomes than did flap-off epi-LASIK, but there was no significant difference between the outcomes of the two procedures, 1 week postoperatively.

## Background

The refractive error of myopia is commonly corrected by eyeglasses, contact lens, implantable contact lens [1], and corneal refractive surgery [2]. In the early 1990s, photorefractive keratectomy (PRK) was first introduced for the surgical correction of myopia [3]; laser ablation refractive surgery was widely applied for anterior segment operation. With advances in the techniques used for epithelium removal, femtosecond laser-assisted in situ keratomileusis (femto-LASIK) and epipolis LASIK (epi-LASIK) have emerged as innovative approaches in the field of refractive surgery.

Depending on whether it was performed with or without flap creation using a microkeratome, the epi-LASIK technique is divided into two types: flap-on and flap-off technique. Ang RE et al. [4] and Zhang Y et al. [5] reported that flap-off epi-LASIK with mitomycin C (MMC) results in lesser pain and corneal haze, and faster visual recovery, while visual results, refractive outcomes, contrast sensitivity (CS), and higher-order aberrations (HOAs) were comparable with those of flap-on epi-LASIK.

Numerous studies have compared the visual outcomes of femto-LASIK and flap-on epi-LASIK (flap creation using a microkeratome). Greater corneal backscattering [6], faster recovery of corneal sensation, lesser degree of spherical aberration (SA), and some CS values [7], and superior outcomes of visual acuity were observed in an early stage [8] after femto-LASIK compared to flap-on epi-LASIK. However, Kezirian GM et al. [9] reported that femto-LASIK and flap-on epi-LASIK were associated with equivalent visual outcomes during the first 3 months postoperative period. Wen D et al. [2] performed a network meta-analysis to compare visual outcomes and quality between these two techniques and found that there were no statistically significant differences in either visual outcomes (efficacy and safety) or visual quality (HOAs and CS); however, they reported that the outcome of femto-LASIK was more predictable than any other type of surgery. Moreover, in the current study, the outcomes were evaluated by Pentacam, which uses a Scheimpflug camera to determine the corneal tomography and topography, thereby providing more detailed corneal biomechanical information [10–12].

The aim of the present study was to compare the visual outcomes and corneal biomechanical properties changes between femto-LASIK and flap-off epi-LASIK.

## Methods

### Patients

A total of 27 patients (40 eyes) who underwent LASIK surgery between April 2014 and February 2016 in the Department of Ophthalmology, Catholic University, St. Mary’s Hospital, Seoul, Korea, were enrolled in this retrospective case series study. This study protocol followed the guidelines of the Declaration of Helsinki and was approved by the Institutional Review Board of St. Mary’s Hospital, Seoul, Korea. Written informed consent was obtained from all patients before commencement of the study.

Patients included in the study underwent refractive surgery for the correction of myopia and had normal preoperative topography. All patients demonstrated at least 1 year of stable refraction before undergoing refractive surgery and were followed-up for at least 2 years postoperatively. Exclusion criteria included the presence of ocular pathology; retinal disorders; previous ocular surgery; co-morbidities, such as diabetes, autoimmune pathologies, and endocrine pathologies; dry eye symptoms; and insufficient follow-up. We also excluded patients with corneal instability, haze or other complications and those undergoing retreatment. The included patients were required to discontinue the use of soft contact lenses for at least 2 weeks and the use of rigid gas permeable lenses for at least 4 weeks prior to surgery.

### Preoperative assessment

All patients underwent a standard ophthalmologic examination preoperatively. The investigations included manifest refraction (MR), cycloplegic refraction, slit-lamp examination, ultrasound pachymetry, dilated funduscopy, and intraocular pressure (IOP) measurement using a Goldmann applanation tonometer. Uncorrected distance visual acuity (UDVA) and corrected distance visual acuity (CDVA) were assessed using Snellen charts. CDVA was assessed using trial frames rather than contact lenses.

Corneal asphericity (Q-value), corneal HOAs, and keratometry were evaluated using a Pentacam (OCULUS Optikgerate GmbH, Wetzlar, Germany). Corneal topography and HOAs were measured using videokeratoscopy (Keratron Scout topographer, Optikon 2000 SpA, Rome, Italy) under photopic conditions (270 lux), which were similar to those used for deciding a surgical plan under an operating microscope.

### Postoperative evaluation

Patients were reviewed at 1 day, 1 week, and 1, 3, and 6 months, and 1 and 2 years postoperatively. All postoperative follow-up visits included the assessment of UDVA, CDVA, and MR assessments, as well as the recording of keratometry readings using a manual keratometer. Pentacam was used to evaluate keratometry, anterior chamber depth (ACD), central corneal thickness (CCT), corneal asphericity (Q-value), and corneal HOAs.

### Surgical procedure

All surgeries were targeted toward achieving emmetropia, and the treatment plan followed the Custom Ablation Manager protocol. Ablations were performed using the AMARIS 750S excimer laser (SCHWIND Eye-Tech Solutions, Kleinostheim, Germany). The aberration-free mode was used, in which ablation was performed with an optimized aspheric profile [13]. All surgeries were performed by a single experienced surgeon (CKJ). Topical anesthetic eye drops containing proparacaine (Alcaine, Alcon-Couvreur, Puur, Belgium) were administered. Femto-LASIK flaps were cut using the iFS Advanced Femtosecond Laser (Abbott Medical Optics, Inc., Irvine, CA, USA) with superior hinges, 100-μm flap thickness, and 8.4- or 8.5-mm flap diameters. Flap-off epi-LASIK was performed using the Epi-K™ epikeratome (Moria SA, Antony, France). After lifting the flap, ablation was performed on a 6.5-mm-diameter optical zone. The planned refractive correction (6.7–9.0 mm) of the ablation zone was carried out automatically in a variable transition zone size. MMC (0.02%) was placed on the residual bed, after which the stromal surface was irrigated with a balanced salt solution, and a bandage contact lens (Senofilcon A, Acuvue Oasys; Johnson & Johnson, Jacksonville, FL, USA) was placed over the surgical site.

The patients were administered topical antibiotic eye drops 4 times/week, topical corticosteroid eye drops 4 times/day (tapered off over 1 week), and topical lubricants.

### Statistical analysis

Data were entered into an Excel spreadsheet database (Microsoft, Redmond, WA, USA), and statistical analysis was performed using SPSS for Windows, version 18.0 (SPSS, Inc., Chicago, IL, USA). Normality of data distribution was tested using the Shapiro-Wilk test. The Wilcoxon rank-sum test and Mann-Whitney U test were used for nonparametric analysis. *P*-values of < 0.05 were considered significant.

## Results

Forty eyes of 27 patients were divided into two groups based on whether a flap was created by femtosecond laser during surgery (20 eyes, femto-LASIK) or not (20 eyes, flap-off epi-LASIK). The characteristics of the two groups are summarized in Table 1. There were no significant differences in the baseline ophthalmic characteristics between both groups.

**Table 1:** Preoperative parameters between the two groups

Parameter	Mean ± Standard Deviation		*P*-value
	femto-LASIK	flap-off epi-LASIK	
SE (D)	−5.94 ± 2.23	−5.94 ± 1.92	0.783
K_1_ (D)	42.35 ± 2.06	42.50 ± 2.13	0.829
K_2_ (D)	43.53 ± 1.31	43.79 ± 2.20	0.989
AD (μm)	100.15 ± 34.13	90.31 ± 27.57	0.813
ACD (mm)	3.12 ± 0.26	3.25 ± 0.30	0.331
RBT (μm)	365.00 ± 43.28	331.95 ± 40.03	0.777
CCT (μm)	597.15 ± 27.69	552.15 ± 28.76	0.597

*femto-LASIK* femtosecond laser-assisted in situ keratomileusis; *epi-LASIK* epipolis laser-assisted in situ keratomileusis; *SE* spherical equivalent; *D* diopters; *K*_*1*_ flat keratometry; *K*_*2*_ steep keratometry; *AD* ablation depth; *ACD* anterior chamber depth; *RBT* preoperative predict residual bed thickness; *CCT* central corneal thickness

Table 2 shows the comparative evaluation of the pre- and postoperative changes between the two groups. There were no significant differences between the two groups with regard to the flattest keratometry reading (K_1_), steepest keratometry reading (K_2_), CCT, or Q-value (Ant. and Post.). Differences between pre- and postoperative K_1_, K_2_, CCT, and Q-value (Ant.) were significant for both the groups (all *P* < 0.05 in femto-LASIK; all *P* < 0.001 in flap-off epi-LASIK).

**Table 2:** Comparison of preoperative and postoperative changes in corneal biometric parameters between the two groups

Parameter	Mean ± Standard Deviation		*P*-value
	femto-LASIK	flap-off epi-LASIK	
K_1_ (D)			
Pre-op	42.65 ± 1.25	42.81 ± 2.09	0.828
Post-op	37.95 ± 2.52	38.04 ± 2.33	0.692
*P*-value	0.001	< 0.001	
K_2_ (D)			
Pre-op	43.79 ± 1.47	43.84 ± 2.11	0.766
Post-op	38.74 ± 2.73	38.61 ± 2.40	0.942
*P*-value	0.003	< 0.001	
CCT (μm)			
Pre-op	597.15 ± 27.69	552.15 ± 28.76	0.597
Post-op	475.27 ± 28.89	454.89 ± 43.54	0.086
*P*-value	< 0.001	< 0.001	
ACD (mm)			
Pre-op	3.06 ± 0.24	3.28 ± 0.30	0.056
Post-op	2.98 ± 0.22	3.19 ± 0.28	0.066
*P*-value	0.001	< 0.001	
Q-value (Ant.)			
Pre-op	−0.41 ± 0.13	−0.39 ± 0.18	0.732
Post-op	0.88 ± 0.65	0.73 ± 0.33	0.732
*P*-value	0.001	< 0.001	
Q-value (Post.)			
Pre-op	−0.30 ± 0.11	−0.30 ± 0.08	0.304
Post-op	−0.28 ± 0.10	−0.28 ± 0.09	0.231
*P*-value	0.068	0.337	

*femto-LASIK* femtosecond laser-assisted in situ keratomileusis; *epi-LASIK* epipolis laser-assisted in situ keratomileusis; *K*_*1*_ flattest keratometry reading; *D* diopters; *K*_*2*_ steepest keratometry reading; *CCT* central corneal thickness; *Pre-op* preoperative; *Post-op* postoperative; *ACD* anterior chamber depth (between endothelium to anterior lens surface); *Ant.* anterior corneal surface; *Post.* posterior corneal surface; *Q-value* corneal asphericity

Changes in the corneal thickness spatial profile (CTSP) are shown in Table 3. There were no statistically significant differences in preoperative and postoperative CTSP values between the two groups at corneal ring diameters of 0-mm, 2-mm, 4-mm, and 8-mm (all *P* > 0.05); however, it was significantly thinner in flap-off epi-LASIK than femto-LASIK at a ring diameter of 6-mm (*P* = 0.039) after surgery. Further details are shown in Table 3.

**Table 3:** Comparison of preoperative and postoperative changes in CTSP between the two groups

Parameter	Mean ± Standard Deviation		*P*-value
	femto-LASIK	flap-off epi-LASIK	
0 mm			
Pre-op	574.45 ± 28.45	547.45 ± 28.34	0.381
Post-op	473.53 ± 28.38	452.47 ± 43.15	0.074
*P*-value	< 0.001	< 0.001	
2 mm			
Pre-op	584.30 ± 28.15	557.20 ± 27.87	0.418
Post-op	490.67 ± 26.29	469.95 ± 42.37	0.068
*P*-value	< 0.001	< 0.001	
4 mm			
Pre-op	614.90 ± 28.67	552.15 ± 28.76	0.431
Post-op	546.53 ± 20.97	454.89 ± 43.54	0.066
*P*-value	< 0.001	< 0.001	
6 mm			
Pre-op	668.95 ± 30.15	639.90 ± 27.30	0.531
Post-op	634.93 ± 20.40	605.47 ± 49.14	0.039
*P*-value	< 0.001	< 0.001	
8 mm			
Pre-op	752.40 ± 31.73	722.95 ± 31.84	0.889
Post-op	731.20 ± 27.52	709.42 ± 41.02	0.074
*P*-value	0.007	0.001	

*femto-LASIK* femtosecond laser-assisted in situ keratomileusis; *epi-LASIK* epipolis laser-assisted in situ keratomileusis; *CTSP* corneal thickness spatial profile; *Pre-op* preoperative; *Post-op* postoperative

**Fig. 1:**
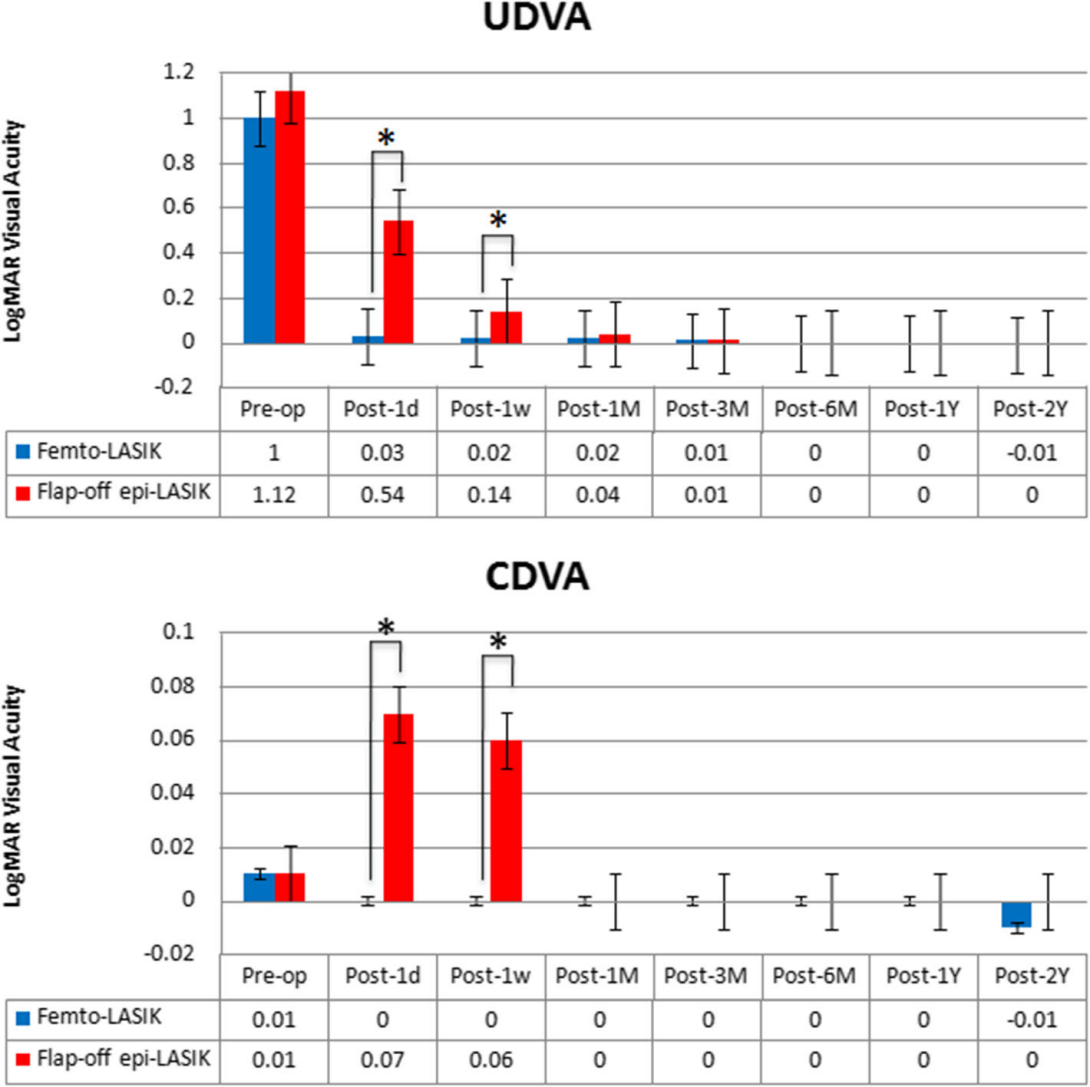
UDVA and CDVA before and after femto-LASIK and flap-off epi-LASIK treatments

The changes in UDVA and CDVA are shown in Fig. 1. The mean changes in logarithm of the minimum angle of resolution (logMAR) UDVA (improvement) were significant in both groups, 2 years postoperatively: from 1.00 ± 0.31 logMAR to − 0.01 ± 0.02 logMAR in femto-LASIK and from 1.12 ± 0.45 logMAR to 0.00 ± 0.00 logMAR in flap-off epi-LASIK (all *P* < 0.001). The improvement was more significant for femto-LASIK at 1 day (0.03 ± 0.06 logMAR in femto-LASIK and 0.54 ± 0.31 logMAR in flap-off epi-LASIK) and 1 week postoperatively (0.02 ± 0.05 logMAR in femto-LASIK and 0.14 ± 0.13 logMAR in flap-off epi-LASIK) (*P* < 0.001 and *P* = 0.019). There were statistically significant differences in CDVA between femto-LASIK and flap-off epi-LASIK at 1 day (0.00 ± 0.00 logMAR in femto-LASIK and 0.07 ± 0.14 logMAR in flap-off epi-LASIK, *P* = 0.026) and 1 week postoperatively (0.00 ± 0.00 logMAR in femto-LASIK and 0.06 ± 0.08 logMAR in flap-off epi-LASIK, *P* = 0.009).

**Fig. 2:**
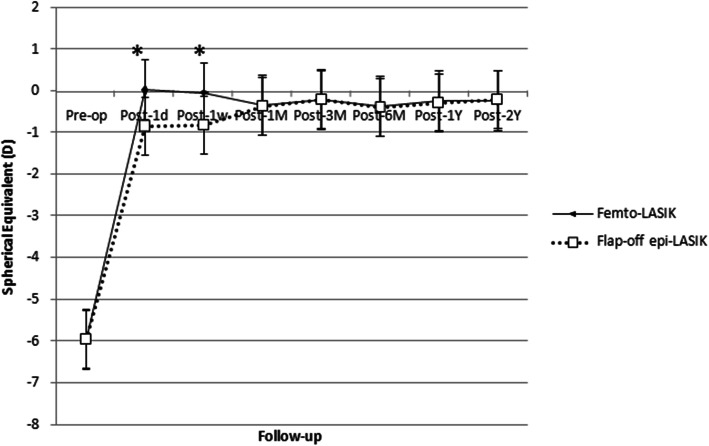
Spherical equivalent refraction measured preoperatively (Pre-op) and at 1 day (d), 1 week (w), 1, 3, 6 months (M), 1 and 2 years (Y) postoperatively (Post-op) between femto-LASIK and flap-off epi-LASIK

The mean preoperative spherical equivalent refraction values were − 5.94 ± 2.23 D (femto-LASIK) and − 5.94 ± 1.62 D (flap-off epi-LASIK), respectively (*P* = 0.904). The postoperative refraction showed significantly higher myopic refraction errors in flap-off epi-LASIK group than femto-LASIK at 1 day (0.03 ± 0.52 D in femto-LASIK, and − 0.84 ± 0.77 D in flap-off epi-LASIK, *P* < 0.001) and 1 week postoperatively (− 0.04 ± 0.56 D in femto-LASIK, and − 0.81 ± 0.98 D in flap-off epi-LASIK, *P* = 0.009), and there were statistically significant improvements in refraction errors in both groups from 1 day after refractive surgery (all *P* < 0.001) (Fig. 2).

Table 4 and Table 5 show the changes in HOAs of the front, back, and total cornea in femto-LASIK and flap-off epi-LASIK. There was a significant reduction in vertical coma (Z_3,-1_) aberration (from − 0.086 ± 0.251 μm to − 0.393 ± 0.335 μm), horizontal secondary astigmatism (Z_4,2_) aberration (from 0.013 ± 0.051 μm to − 0.113 ± 0.113 μm), and induction of SA (Z_4,0_) (from 0.271 ± 0.132 μm to 0.479 ± 0.139 μm) between pre- and post-femto-LASIK in the front corneal HOAs (*P* = 0.021, *P* = 0.001, and *P* = 0.001, respectively). In terms of total corneal HOAs changes, there was a significant reduction in vertical coma (Z_3,-1_) aberration (from − 0.128 ± 0.215 μm to − 0.368 ± 0.328 μm), horizontal secondary astigmatism (Z_4,2_) aberration (from − 0.007 ± 0.055 μm to − 0.122 ± 0.117 μm), and induction of SA (Z_4,0_) (from 0.168 ± 0.061 μm to 0.430 ± 0.137 μm) between pre- and post-femto-LASIK (*P* = 0.007, *P* = 0.004, and *P* < 0.001, respectively). However, in terms of back corneal HOAs changes, there was a significant induction of vertical coma (Z_3,-1_) aberration, (from 0.013 ± 0.025 μm to 0.027 ± 0.027 μm), reduction of oblique trefoil (Z_3,-3_) aberration (from − 0.026 ± 0.042 μm to − 0.055 ± 0.037 μm), and oblique tetrafoil (Z_4,-4_) aberration (from 0.006 ± 0.030 μm to − 0.008 ± 0.029 μm) between pre- and post-femto-LASIK (*P* = 0.015, *P* = 0.046, and *P* = 0.049, respectively). In flap-off epi-LASIK, there was only significant induction of SA (from 0.250 ± 0.128 μm to 0.626 ± 0.232 μm, and from − 0.156 ± 0.033 μm to 0.556 ± 0.227 μm) between pre- and postoperative in the front and total corneal HOAs (all *P* < 0.001). In the back corneal HOAs, there was a significant induction of horizontal secondary astigmatism (Z_4,2_) aberration (from − 0.001 ± 0.016 μm to 0.007 ± 0.018 μm) and reduction of SA (Z_4,0_) (from − 0.156 ± 0.033 μm to − 0.163 ± 0.037 μm) between pre- and postoperative periods (*P* = 0.027 and *P* = 0.011).

**Table 4:** Comparison of preoperative and postoperative changes in corneal HOAs in femto-LASIK at 6-month postoperatively

Parameter	Mean ± Standard Deviation		*P*-value
	Preoperative	Postoperative	
Front corneal HOAs			
Z_3,3_	0.006 ± 0.076	−0.017 ± 0.149	0.644
Z_3,1_	0.007 ± 0.139	−0.019 ± 0.471	0.845
Z_3,-1_	−0.086 ± 0.251	−0.393 ± 0.335	0.021
Z_3,-3_	−0.056 ± 0.120	0.026 ± 0.176	0.206
Z_4,4_	−0.024 ± 0.090	−0.069 ± 0.073	0.233
Z_4,2_	0.013 ± 0.051	−0.113 ± 0.113	0.001
Z_4,0_	0.271 ± 0.132	0.479 ± 0.139	0.001
Z_4,-2_	−0.016 ± 0.047	0.005 ± 0.089	0.479
Z_4,-4_	0.007 ± 0.076	0.034 ± 0.126	0.496
Back corneal HOAs			
Z_3,3_	0.008 ± 0.048	0.009 ± 0.052	0.971
Z_3,1_	−0.001 ± 0.025	0.004 ± 0.035	0.463
Z_3,-1_	0.013 ± 0.025	0.027 ± 0.027	0.015
Z_3,-3_	−0.026 ± 0.042	−0.055 ± 0.037	0.046
Z_4,4_	−0.038 ± 0.035	−0.041 ± 0.038	0.695
Z_4,2_	−0.009 ± 0.014	−0.008 ± 0.012	0.695
Z_4,0_	−0.143 ± 0.017	−0.140 ± 0.024	0.277
Z_4,-2_	0.003 ± 0.013	−0.001 ± 0.016	0.339
Z_4,-4_	0.006 ± 0.030	−0.008 ± 0.029	0.049
Total corneal HOAs			
Z_3,3_	0.042 ± 0.114	−0.008 ± 0.157	0.339
Z_3,1_	−0.001 ± 0.131	−0.015 ± 0.450	0.878
Z_3,-1_	−0.128 ± 0.215	−0.368 ± 0.328	0.007
Z_3,-3_	−0.031 ± 0.122	−0.023 ± 0.173	0.883
Z_4,4_	−0.096 ± 0.091	−0.108 ± 0.064	0.659
Z_4,2_	−0.007 ± 0.055	−0.122 ± 0.055	0.004
Z_4,0_	0.168 ± 0.061	−0.430 ± 0.137	< 0.001
Z_4,-2_	−0.016 ± 0.047	0.004 ± 0.098	0.538
Z_4,-4_	0.002 ± 0.089	0.033 ± 0.133	0.423

*femto-LASIK* femtosecond laser-assisted in situ keratomileusis; *HOAs* higher-order aberrations

**Table 5:** Comparison of preoperative and postoperative changes in corneal HOAs in flap-off epi-LASIK at 6-month postoperatively

Parameter	Mean ± Standard Deviation		*P*-value
	Preoperative	Postoperative	
Front corneal HOAs			
Z_3,3_	0.020 ± 0.076	0.033 ± 0.157	0.702
Z_3,1_	0.022 ± 0.151	−0.019 ± 0.476	0.666
Z_3,-1_	−0.082 ± 0.229	−0.191 ± 0.303	0.128
Z_3,-3_	−0.059 ± 0.104	−0.004 ± 0.204	0.217
Z_4,4_	−0.020 ± 0.084	−0.021 ± 0.091	0.975
Z_4,2_	0.000 ± 0.051	−0.060 ± 0.173	0.141
Z_4,0_	0.250 ± 0.128	0.626 ± 0.232	< 0.001
Z_4,-2_	−0.015 ± 0.044	−0.014 ± 0.088	0.947
Z_4,-4_	0.007 ± 0.070	0.006 ± 0.146	0.975
Back corneal HOAs			
Z_3,3_	0.001 ± 0.063	0.000 ± 0.063	0.968
Z_3,1_	−0.006 ± 0.026	−0.006 ± 0.039	0.913
Z_3,-1_	−0.002 ± 0.035	0.001 ± 0.034	0.464
Z_3,-3_	−0.028 ± 0.041	−0.023 ± 0.054	0.639
Z_4,4_	−0.035 ± 0.027	−0.041 ± 0.028	0.147
Z_4,2_	−0.001 ± 0.016	0.007 ± 0.018	0.027
Z_4,0_	−0.156 ± 0.033	−0.163 ± 0.037	0.011
Z_4,-2_	−0.004 ± 0.011	−0.005 ± 0.014	0.796
Z_4,-4_	0.013 ± 0.025	0.015 ± 0.026	0.713
Total corneal HOAs			
Z_3,3_	0.020 ± 0.063	0.035 ± 0.180	0.692
Z_3,1_	0.015 ± 0.026	−0.026 ± 0.456	0.653
Z_3,-1_	−0.077 ± 0.035	−0.185 ± 0.302	0.179
Z_3,-3_	−0.084 ± 0.041	−0.024 ± 0.216	0.211
Z_4,4_	−0.053 ± 0.027	−0.059 ± 0.091	0.815
Z_4,2_	−0.004 ± 0.016	−0.056 ± 0.171	0.201
Z_4,0_	0.194 ± 0.033	0.556 ± 0.227	< 0.001
Z_4,-2_	−0.019 ± 0.011	−0.018 ± 0.095	0.959
Z_4,-4_	0.019 ± 0.025	0.021 ± 0.137	0.955

*epi-LASIK* epipolis laser-assisted in situ keratomileusis; *HOAs* higher-order aberrations

**Fig. 3:**
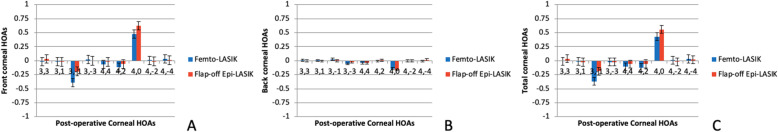
Comparison of changes in the corneal higher-order aberrations (HOAs) between femto-LASIK and flap-off epi-LASIK. **a.** The differences in postoperative corneal HOAs between femto-LASIK and flap-off epi-LASIK in the front cornea. **b.** The differences in postoperative corneal HOAs between femto-LASIK and flap-off epi-LASIK in the back cornea. **c.** The differences in postoperative corneal HOAs between femto-LASIK and flap-off epi-LASIK in the total cornea

When we compared the postoperative corneal HOA changes between the two groups, the increment in SA (Z_4,0_) was higher in flap-off epi-LASIK than femto-LASIK: 0.626 ± 0.232 μm and 0.479 ± 0.139 μm in the front cornea, 0.556 ± 0.227 μm and 0.430 ± 0.137 μm in the total cornea, respectively (*P* = 0.016 and *P* = 0.017). With regard to the back corneal HOAs, there were significant differences in vertical coma (Z_3,-1_) aberration: 0.027 ± 0.027 μm (femto-LASIK) and 0.001 ± 0.034 μm (flap-off epi-LASIK); horizontal secondary astigmatism (Z_4,2_) aberration: − 0.008 ± 0.012 μm (femto-LASIK) and 0.007 ± 0.018 μm (flap-off epi-LASIK); oblique tetrafoil (Z_4,-4_) aberration: − 0.008 ± 0.029 μm (femto-LASIK) and 0.015 ± 0.026 μm (flap-off epi-LASIK), respectively (*P* = 0.018, *P* = 0.007, and *P* = 0.022, respectively) (Fig. 3).

## Discussion

Many studies have investigated whether flap creation using a femtosecond laser (femto-LASIK) is more effective than that using a microkeratome (flap-on epi-LASIK) [6–9]. However, in the present study, we compared the outcomes between femto-LASIK and flap-off epi-LASIK. Previously, Kalyvianaki MI et al. [14] reported that flap-on epi-LASIK and flap-off epi-LASIK produced equivalent visual and refractive results for the treatment of low and moderate myopia. Furthermore, Na KS et al. [15] found that flap-off epi-LASIK yielded superior visual recovery and corneal re-epithelialization than flap-on epi-LASIK surgery in the early postoperative period.

Corneal haze with decreased corneal transparency is typically determined by corneal backward light scattering. It has been reported that ablation volume may increase the degree of backscattering [16], and cases of severe myopia that require more ablation may require a higher dose of MMC during the refractive procedure [17, 18]. Sia RK et al. [19] and Chen J et al. [20] reported that MMC was beneficial for the reduction of corneal haze, without delaying epithelialization. The present study demonstrated little difference between the two techniques. Significantly better visual and refractive outcomes were associated with femto-LASIK than flap-off epi-LASIK at 1 day and 1 week postoperatively, with no additional significant differences during the remaining follow-up.

Myopic or hyperopic refractive surgery aims to correct the corneal shape by changing the keratometric power [4, 21]. Huang J et al. [22] and Jain R et al. [23] obtained high degree of repeatability for corneal curvatures after LASIK using a Scheimpflug camera, with no significant difference between the automatic and manual keratometric readings [24]. In this study, we used the Scheimpflug camera to evaluate the outcomes after refractive surgery. We found that both procedures showed a statistically significant decrease in CCT, keratometry readings, and ACD values after surgery. Dai ML and associates [25] reported that the ACD was shallower in LASIK than in non-operated myopic eyes.

The surface ablation technique can help avoid numerous surgical complications arising from the creation of a lamellar corneal flap required in LASIK, and can theoretically provide more stable corneal biomechanics. Shih PJ et al. [26] demonstrated corneal biomechanical simulation of stress concentration after refractive surgery, and they proposed that both surface and stromal ablation techniques caused stress in an obliquely downwards direction after surgery. We postulated that these changes of corneal biomechanical properties may influence the changes in corneal SA after corneal refractive surgery.

The concept of CTSP was first introduced by Ambrosio R Jr. et al. [27]. Furthermore, Buhren J et al. [28] found that the posterior aberrations and thickness spatial profile data did not markedly improve discriminative ability over that of anterior wavefront data alone. In our study, we used CTSP to evaluate changes in corneal thickness at different corneal diameters. We found that CTSP changes were significantly smaller in flap-off epi-LASIK than femto-LASIK at a corneal ring diameter of 6-mm; the CTSP changes in the central region were greater than that at the mid-periphery. In addition, the corneal HOAs at the 6.5-mm diameter were significantly different in the front and total HOAs of SA, while few significant differences were found in posterior HOAs of vertical coma aberration, oblique trefoil aberration, and oblique tetrafoil aberration. We postulated that these changes in CTSP may influence the changes in corneal HOAs, and may also affect the Q-value (8 mm) changes after LASIK, in a manner dependent on the size of the optical zone being treated.

The effect of SA on the depth of focus has been investigated using adaptive optics systems [29]. The depth of focus, by definition, is relatively insensitive to focal length and subject distance for a fixed f-number. Typically, myopia is a condition in which light is focused in front of the retina rather than on it. However, corneal refractive surgery is a type of refractive surgery that ablates the corneal tissue to change the accommodation power. Wallace HB et al. [30] found that ACD was significantly reduced by 0.10 mm with accommodation, and statistically significant changes in corneal curvatures were seen in all participants with accommodation.

The principle of refractive surgery is to induce positive SA shifts for the correction of myopia, and negative shifts for hyperopic correction [31, 32]. Moreover, the concept of the SCHWIND Amaris 750S excimer laser involves using the optimized aspheric profile [13] to prevent surgically induced HOAs, especially SA and coma aberration. Although the amount of corneal SA and asphericity are intrinsically related, they provide a 2:1 correspondence between corneal and ocular SA [33]. However, in the present study, there was significant increment in SA: 0.479 ± 0.139 μm in femto-LASIK and 0.626 ± 0.232 μm in flap-off epi-LASIK, and the logMAR UDVA achieved − 0.01 ± 0.02 logMAR in femto-LASIK and 0.00 ± 0.00 logMAR in flap-off epi-LASIK at 2 years postoperatively.

Total corneal refractive power involves compensation for negative posterior refractive power by positive anterior refractive power. Steepening of the anterior corneal surface increases the positive refractive power; when both surfaces bulge similarly, the anterior surface induces far greater absolute refractive changes than the posterior surface. According to our results, the patterns of corneal HOA changes were similar, while changes in front and total corneal HOAs were significantly different after both corneal refractive surgeries.

The induced changes in corneal asphericity (Q-value) and SA after laser ablation are key factors associated with the selection of candidates for refractive surgery. Scheimpflug imaging provided reliable measurements, consistent with those reported in the literature; there was a positive change in the Q-value of the anterior surface after myopic ablation and a negative change after hyperopic ablation [34].

Corneal aberrations are usually positive, aberrations of the lens are usually negative, and the total SA changes more than other HOAs with accommodation. Moreover, ocular wavefront aberrations are primarily created in the cornea and lens, and are strongly affected by several factors, including the accommodative state [35], pupil diameter [36], tear film [37], age [38], and pupil entrance decentration [39]. We found a statistically significant difference in postoperative SA between the two different surgical techniques, but found no clinically significant difference up to 2 years postoperatively; femto-LASIK produced superior visual outcomes to flap-off epi-LASIK in the early postoperative stage.

A meta-analysis shows that there were no statistically significant differences in either visual outcomes or visual quality between different corneal refractive surgery techniques, and that femto-LASIK shows a better predictability than any other type of surgery. However, this study was limited by the small sample size; therefore, studies involving a larger population of patients are necessary to ensure more dependable results [40].

## Conclusion

Refractive surgery has been regarded as an excellent surgical option, negating the need for contact lenses or glasses. Our study results indicated that both femto-LASIK and flap-off epi-LASIK were safe, effective, and predictable refractive surgeries. Femto-LASIK would be a better surgical option that provides lesser postoperative SA after surgery and superior visual outcomes in the early postoperative stage. Preoperative corneal thickness should be considered when choosing corneal refractive surgery in clinical practice.

## Data Availability

The datasets obtained and/or analyzed during the current study are available from the corresponding author on reasonable request.

## Abbreviations

ACD: Anterior chamber depth
AD: Ablation depth
CCT: Central corneal thickness
CDVA: Corrected distance visual acuity
CS: Contrast sensitivity
Epi-LASIK: Epipolis laser-assisted in situ keratomileusis
Femto-LASIK: Femtosecond laser-assisted in situ keratomileusis
HOAs: Higher-order aberrations
K_1_: Flattest keratometry reading
K_2_: Steepest keratometry reading
MR: Manifest refraction
Q-value: Corneal asphericity
RBT: Preoperative predict residual bed thickness
UDVA: Uncorrected distance visual acuity
